# Pharmacokinetics, Tissue Distribution, and Metabolism Study of Icariin in Rat

**DOI:** 10.1155/2017/4684962

**Published:** 2017-11-13

**Authors:** Shunjun Xu, Jiejing Yu, Jingjing Zhan, Liu Yang, Longgang Guo, Yijuan Xu

**Affiliations:** ^1^Guangzhou ImVin Pharmaceutical Co., Ltd., Guangzhou 510663, China; ^2^Guangdong Provincial Hospital of Chinese Medicine, Guangzhou University of Chinese Medicine, Guangzhou 510120, China

## Abstract

Icariin is one of the predominant flavonoids contained in Herba Epimedii (Yin-yang-huo in Chinese), a well-known Chinese medicine for the treatment of cancers and immune system diseases. Although Herba Epimedii has been widely used in China and there are so many and various research reports on the herbal drug and its main flavones, very limited data is available on the tissue distribution and biotransformation of icariin. In the present study, a liquid chromatographic method combined with electrospray ionization tandem mass spectrometry was developed to quantify the concentration of icariin in rat plasma and various tissues collected at different time points after oral administration of the total flavonoid extract of Herba Epimedii at a dose of 0.69 g/kg (corresponding to 42 mg/g icariin). Biological samples were processed by simple protein precipitation. Genistein was chosen as internal standard. The method was successfully applied to plasma pharmacokinetic and tissue distribution studies of icariin in rat. As a result, it was worth noting that the tissue distribution characteristics of icariin exhibited a significant gender difference. Moreover,* in vivo* metabolism of icariin was also investigated. A total of 11 potential metabolites were found in rat feces collected in different time periods after oral and intramuscular administration of icariin.* In vivo* metabolic pathways were involved in hydrolysis, demethylation, oxidation, and conjugation. The preclinical data would be useful for fully understanding* in vivo* disposition of this compound and interpreting the mechanism of its biological response.

## 1. Introduction

Herba Epimedii, the fresh or dried aerial parts of* Epimedium* genus, has been used not only as a kind of food but also as a Chinese herbal drug for thousands of years. It is officially listed in all versions of Chinese pharmacopoeia with the name “Yin-yang-huo,” which originates from 4 species of* Epimedium*, that is,* E. brevicornum* Maxim.,* E. sagittatum* Maxim.,* E. pubescens* Maxim., and* E. koreanum* Nakai. Herba Epimedii possesses estrogen-like activity and prevention of osteoporosis [1–3], improvement of sexual function [4, 5], regulation of immune response [6, 7], and putative anticancer activity [8]. Icariin is the most abundant bioactive component of this herbal drug. There has been a lot of literature showing that icariin has various biological activities such as anticancer and immune regulation, prevention and treatment of osteoporosis, prolongation of lifespan, and improvement of sexual function [9–14]. Therefore, icariin has received more and more attention from medical and pharmaceutical researchers.

Despite icariin displaying a wide variety of bioactivities, it has been reported that this compound has very low oral bioavailability. Chen et al. [15] reported that prenylated flavonoids in Herba Epimedii, such as icariin, epimedin A, epimedin B, and epimedin C, should be hydrolyzed into secondary glycosides in small intestine prior to absorption. Our study [16] also demonstrated that the recovery of icariin in urine was less than 0.425‰, suggesting that most of icariin should be metabolized and excreted as metabolites. Clearly, it is necessary to investigate the* in vivo* drug disposition of icariin and to identify its metabolites, which is useful for understanding the biological activity of icariin.

The pharmacokinetics of icariin in rat has been previously investigated and reported. Lu et al. [17] developed a UPLC-MS/MS method to quantify icariin in rat plasma after oral administration of a Chinese herbal preparation, and the results indicated that there is no statistic difference in the main pharmacokinetic parameters of icariin between male and female rats. Wang et al. [18] established an HPLC method for the determination of epimedins A, B, and C and icariin in plasma with peak concentrations of 0.45 ± 0.01, 0.53 ± 0.02, 4.11 ± 0.08, and 1.31 ± 0.07 *μ*g/mL, respectively, about 0.25 h after oral administration of Herba Epimedii extract (10 g/kg body weight) to rats. Although these articles separately focused on pharmacological, plasma pharmacokinetic, and metabolism studies of icariin, the systemic* in vivo* disposition, especially tissue distribution and complete metabolic profile of this compound, has not been summarized so far.

In the present study, we established and validated a quantitative LC-MS/MS method of icariin in rat plasma and various tissue homogenate and successfully applied the method to pharmacokinetic and tissue distribution studies of icariin. We also investigated the tissue distribution features of icariin in male and female rats and minutely summarized the similarities and differences of icariin distribution between male and female rats. Moreover, we analyzed the complete metabolic profiles of this compound in rat feces and urine following oral and intramuscular administration. Accordingly, the metabolites of icariin were identified or tentatively characterized and the potential metabolic pathways of icariin were also described in this paper.

## 2. Materials and Methods

### 2.1. Chemicals and Reagents

**Figure 1:**
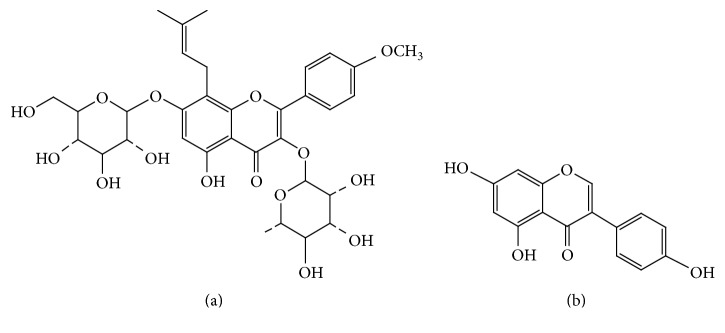
Chemical structure of icariin (a) and genistein (internal standard, b).

Icariin (purity 98%) and genistein (IS, purity 98%) were purchased from the National Institute for the Control of Pharmaceutical and Biological Products (Beijing, China). Icaritin was provided by Shanghai Winherb Medical Technology Co., Ltd. (Shanghai, China). Icariside II, baohuoside II, sagittatoside B, and 2′′-O-rhamnosyl-icariside II were purchased from Baoji Herbest Bio-Tech Co., Ltd. (Baoji, China). Their purities were >98% determined by HPLC-UV analysis. The chemical structures of icariin and IS are shown in Figure 1. Herba Epimedii extract originating from* E. sagittatum* Maxim. was produced and provided by Guangzhou ImVin Pharmaceutical Co., Ltd. (Guangzhou, China). LC grade methanol and acetonitrile were obtained from Fisher Company (Fisher Scientific, Fairlawn, NJ). HPLC-grade formic acid was purchased from Tedia Company, Inc. (Fairfield, OH, USA). Water was purified using a Millipore purification system (Millipore, Milford, MA, USA). All of other chemicals and reagents were of analytical grade and were commercially available.

### 2.2. Chromatographic Conditions

For plasma pharmacokinetic and tissue distribution studies, the LC-MS/MS system consisted of a Shimadzu LC-20A HPLC system and an Applied Biosystems Sciex API 4000^+^ (MDS-Sciex, Concord, Canada) equipped with a Turbo Ion Spray. Data acquisition and processing were performed using Analyst 1.6.3 software (Applied Biosystems, Foster City, CA, USA). Chromatographic separation was carried out on an Agilent Eclipse XDB-C_18_ (2.1 × 150 mm, 5 *μ*m), and the column temperature was maintained at 40°C. The mobile phase was composed of acetonitrile-water containing 0.1% formic acid (32 : 68, v/v). The flow rate was set at 0.3 mL/min. The autosampler was set at 4°C and the injection volume was 5 *μ*L.

In the study on metabolism of icariin, metabolites were identified by LTQ-Orbitrap XL mass spectrometer (Thermo Electron, Bremen, Germany) coupled with an ESI source. An Agilent Eclipse SB-C_18_ column (2.1 × 100 mm, 1.8 *μ*m) was employed and column temperature was maintained at 40°C. Mobile phase was composed of A (0.1% formic acid aqueous solution) and B (acetonitrile) with a gradient elution as follows: 0–35 min, 15–30% B; 35–55 min, 30–50% B; 55–70 min, 50% B; 70–80 min, 50–100% B; 80–90 min, 100% B. The flow rate of mobile phase was 0.2 mL/min. The injection volume of samples was 5 *μ*L.

### 2.3. Mass Spectrometer Conditions

For plasma pharmacokinetic and tissue distribution studies, mass spectrometer was operated in negative mode for the detection of icariin and IS. Quantification was performed with multiple reaction monitoring (MRM) mode, and the fragmentation transition was *m*/*z* 735.7 → 513.5 for icariin ([M+CH_3_COOH-H]^−^) and *m*/*z* 269.0 → 133.2 for IS. ESI-MS/MS operation parameters were as follows: ion spray (IS) voltage, −4500 V; source temperature (TME), 350°C. Gas 1, Gas 2, curtain gas, and collision gas (nitrogen) were separately set at 50, 50, 45, and 12 psi.

To study the metabolism profiles of icariin, optimized operating parameters in negative-ion mode were as follows: sheath gas flow rate, 40 arbitrary units; auxiliary gas flow rate, 5 arbitrary units; electrospray voltage, 3.5 kV; capillary voltage, −32 V; capillary temperature, 270°C.

### 2.4. Standard and Sample Preparation

#### 2.4.1. Preparation of Stock Solutions, Calibration Standards, and Quality Controls

The stock solution (1 mg/mL) of icariin and IS was prepared with methanol, respectively. The stock solution of icariin was diluted with methanol to make a series of working solutions of 5, 50, 500, and 5000 ng/mL. The stock solution of IS was diluted with methanol to make a working solution of 1 *μ*g/mL. All stock and working solutions were kept at −20°C.

A series of calibration standards containing 100 ng/mL of IS were separately prepared to contain icariin concentrations of 1, 2, 5, 10, 20, 50, 100, 200, and 500 ng/mL by freshly spiking the working solution of icariin and IS into blank biological matrix. Quality control (QC) samples at three concentration levels (low: 2 ng/mL; medium: 20 ng/mL; high: 400 ng/mL) were, respectively, prepared in the same way. All of the calibration standards and QC samples were freshly prepared before use.

#### 2.4.2. Sample Preparation

For pharmacokinetic and distribution studies, blank plasma was obtained from drug-free rats (Sprague-Dawley rats). Feces and urine were collected 24 hours before and after the time of administration. Blank tissue homogenate was prepared by homogenizing the tissue of drug-free rat and then diluted with three times the volume of saline solution.

Microcentrifuge tubes containing 15 *μ*L of IS and 150 *μ*L of each calibration standard and the QC samples were dried under a gentle stream of nitrogen. An aliquot of 150 *μ*L of drug-free plasma or blank tissue homogenate was added to these tubes and then followed by a single-step protein precipitation, by adding 600 *μ*L of methanol, vigorously shaking on a vortex-mixer for 10 min, and then centrifuging the tubes at 14,800 rpm for 10 min. The supernatant consisting of the organic solvent was transferred to a fresh tube and dried under nitrogen. The residue was reconstituted in 150 *μ*L of methanol and centrifuged to filter by a microspin filter tube (0.22 *μ*m nylon, Alltech) at 14,800 rpm for 1 min. A 5 *μ*L aliquot of the filtrate was injected into RRLC-MS/MS for analysis.

In the metabolism study, rat feces samples were pulverized with a mortar and pestle. Methanol (1 : 5 w/v) was added and homogenized with the pulverized feces. Mixture was extracted by ultrasound for 90 min and centrifuged at 14,800 rpm for 10 min. The supernatant fluid was transferred to an autosampler vial for analysis. For urine sample, 2 mL of rat urine flowed through a pretreatment C_18_ SPE cartridge with gravity. The solid-phase cartridge was washed with 2 mL water and then eluted with 2 mL methanol. The methanol eluate was evaporated to dryness by water bath at 60°C. Residue was dissolved into 500 *μ*L methanol for analysis. The injection volume of urine sample was 5 *μ*L.

### 2.5. Method Validation

The method was validated in terms of specificity, linearity, sensitivity, precision and accuracy, stability, recovery, and matrix effect.

#### 2.5.1. Specificity

Specificity was assessed by analyzing blank biological samples, blank biological matrix spiked with icariin and IS, and real biological samples from rat after oral administration of the total flavonoid extract of Herba Epimedii.

#### 2.5.2. Linearity and Sensitivity

Calibration curves were obtained by plotting the peak area ratios (*y*-axis) of icariin to IS against the nominal concentration (*x*-axis) of icariin and assessed by weighted least-squares linear regression using 1/*x*^2^ as weighting factor. Concentration of QC and biological samples were calculated using the regression equation of the calibration curve.

LLOQ was defined as the lowest concentration on the standard curve at which the standard deviation was within 20% and accuracy was within 100 ± 20%, and it was established using five samples independent from the standard curve.

#### 2.5.3. Precision and Accuracy

Intraday precision and accuracy were evaluated by analyzing five reduplicate QC samples at three concentrations on the same day. Interday precision and accuracy were established by three analytical batches on three consecutive days. The accuracy was expressed as (observed  concentration/nominal  concentration) × 100%. Intra- and interday precision was obtained by one-way analysis of variance (ANOVA) testing and was expressed as relative standard deviation (RSD). The accuracy was required to be within 85–115%, and the precision should not exceed 15%.

#### 2.5.4. Stability

The stability of icariin was evaluated with quintuplicate spiked biological samples at three QC levels, which were tested under the following conditions: (1) the freeze-thaw stability of icariin in rat biological samples through three freeze-thaw (−80°C to room temperature) cycles; (2) the short-term stability of icariin in rat biological samples at room temperature for 6 h; (3) the long-term stability of icariin in rat biological samples stored at −80°C for 30 days; and (4) the postpreparative stability of icariin during storage in the autosampler at 4°C for 24 h.

#### 2.5.5. Recovery and Matrix Effects

Extraction recovery and matrix effect were determined by comparing the peak areas of analyte between three types of samples: (1) biological samples spiked with known amount of analyte before sample preparation; (2) biological samples spiked with known amount of analyte after sample preparation; (3) pure standard solution of analyte. The difference in peak areas between samples 1 and 2 reflects the extraction recovery, while the difference in peak areas between samples 2 and 3 reflects the matrix effect. The accuracy of the method was calculated by comparing theoretically and experimentally measured analyte levels.

### 2.6. Pharmacokinetic, Distribution, and Metabolism Studies of Icariin

In the present study, the experimentation on rats gained the approval of an independent ethics committee at Guangdong Provincial Hospital of Chinese Medicine. The experiment was performed in an SPF level laboratory, authorized by Guangdong Provincial Government.

#### 2.6.1. Plasma Pharmacokinetics

Six male Sprague-Dawley (SD) rats (250 ± 20 g) were obtained from Guangdong Province Laboratory Animal Center and kept in an environmentally controlled breeding room for 3 days before the experiment started. The rats were orally given a single dose of 0.69 g/kg of total flavonoid extract from Herba Epimedii (corresponding icariin approximately at 42 mg/g). Blood samples were serially withdrawn from rat's retroorbital plexus into heparin lithium-anticoagulant tubes at 0 (before dose), 0.083, 0.25, 0.5, 0.75, 1, 1.5, 2, 3, 4, 6, 8, 10, 12, and 24 h after administration. The collected samples were then centrifuged at 3000 rpm for 15 min and supernatant plasma was transferred into another tube and stored at −80°C until analysis.

#### 2.6.2. Tissue Distribution Study

Thirty-six SD rats (220 ± 20 g, 18 males and 18 females) were orally given a single dose of 0.69 g/kg of total flavonoid extract from Herba Epimedii (approximately containing 42 mg/g of icariin). Animals were euthanized at the time points of 0.25, 0.5, 1, 2, 4, and 6 h after dose (*n* = 6 at each time point). Blood was collected via cardiac puncture (about 1000 *μ*L) and immediately transferred into heparinized tube. The collected sample was centrifuged at 3000 rpm for 15 min and supernatant plasma was transferred into another tube and stored at −80°C until analysis. Nine kinds of rat tissues, including liver, heart, spleen, lung, kidney, brain, testicle, uterus, and ovary, were immediately removed and rinsed with ice-cold saline to remove extraneous blood and blot-dried and then stored in preweighted and labeled vials at −80°C until use. On the day of analysis, the tissue samples were thawed and weighed to obtain the tissue weight expressed as the difference between the pre- and postvial weights.

#### 2.6.3. Metabolism Study of Icariin in Rat Urine and Feces

In this study, six SD rats (220 ± 20 g, male) were individually placed in metabolic cages and halved into two groups at random. The reference standard of icariin (60 mg) was dissolved in 8 mL of sterile water for injection containing 40% 1, 3-propanediol (v/v). The intramuscular group was dosed with icariin (20 mg/kg) by intramuscular injection of this solution. Oral administration of icariin (50 mg/kg) was conducted on the other group. Feces and urine samples were separately collected at 24 h after administration of icariin. All the samples were stored at −80°C until use.

### 2.7. Data Analysis

Plasma concentration versus time profiles was obtained from each individual rat, and the calculation of pharmacokinetic parameters was performed via DAS 2.1.1 software (Mathematical Pharmacology Professional Committee of China, Shanghai, China) with noncompartmental pharmacokinetic analysis. All data were expressed as mean ± standard deviation (SD).

## 3. Results and Discussions

### 3.1. Optimization of Sample Preparation

To obtain better extraction efficiency and less endogenous interference, different extraction procedures, including solid-phase extraction and liquid-liquid extraction with different solvents, were tested. But all the extraction recoveries were not satisfactory. Thus, a simpler and less time-consuming method, protein precipitation with methanol was finally used, which was testified to be rapid, repeatable, and sensitive enough for the determination of biological samples in the present study.

### 3.2. Optimization of Chromatography and Mass-Spectrometric Conditions

It is very important to pick a suitable IS to get high accuracy and precision when a mass spectrometer is equipped with LC as a detector. In this study, genistein was chosen as the IS because of its similar basic structure, physicochemical property, and mass-spectrometric behavior to those of icariin.

**Figure 2:**
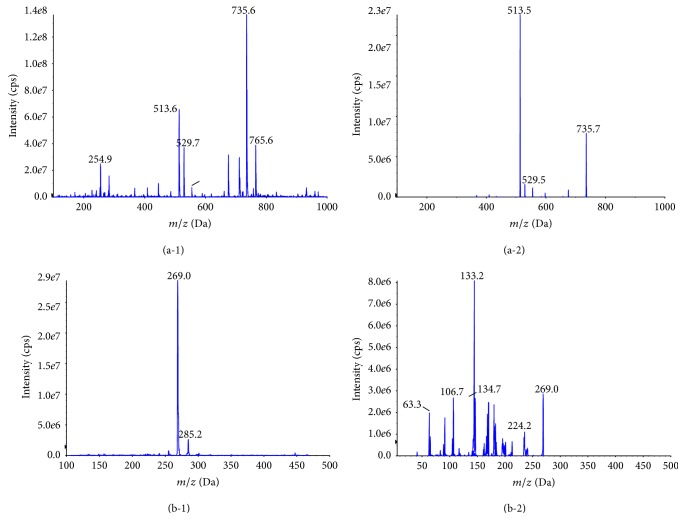
Full scan mass spectrum of icariin (a-1) and internal standard (genistein, b-1); product ion spectrum of icariin (a-2) and internal standard (genistein, b-2).

The mass-spectrometric behavior of icariin and IS was investigated using both positive- and negative-ion ESI. It was found that both icariin and IS had good responses in negative-ion detection mode with low background noise level. Multiple reaction monitoring (MRM) scan type was used to improve specificity. Detection was finally operated in negative-ion mode. Quantification was performed by MRM mode and the selected monitor ion was *m*/*z* 735.7 → 513.5 for icariin ([M+CH_3_COOH-H]^−^) and *m*/*z* 269.0 → 133.2 for IS. The full scan and MS/MS spectra of icariin and IS were shown in Figure 2.

**Figure 3:**
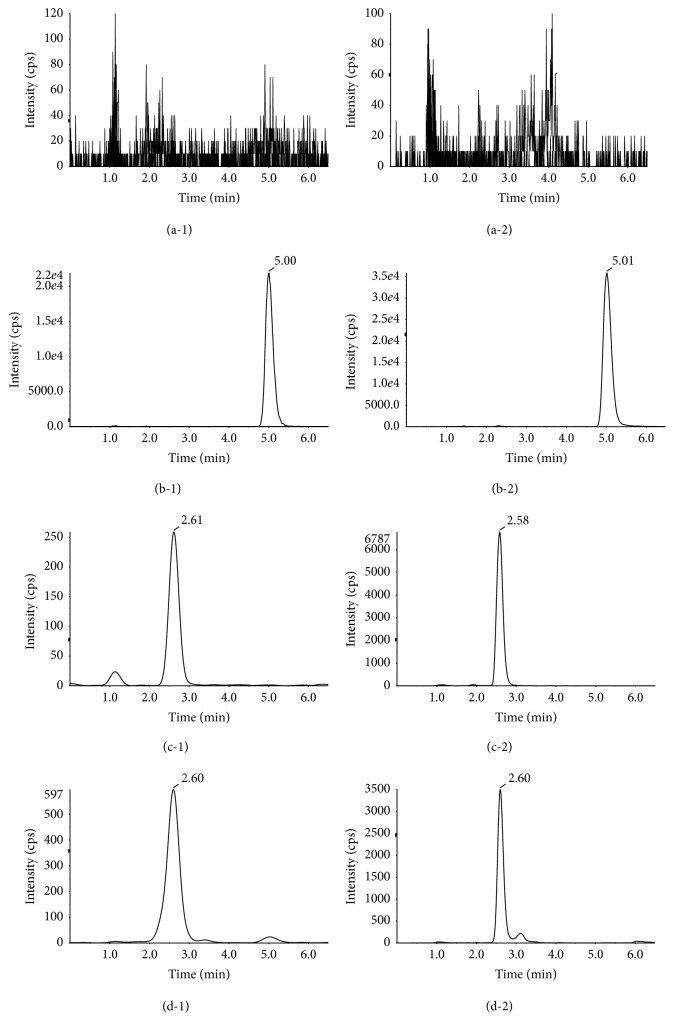
Representative MRM chromatograms of blank plasma (a-1), blank liver (a-2), blank plasma spiked with IS (b-1), blank liver spiked with IS (b-2), blank plasma spiked with icariin at LOQ (c-1), blank liver spiked with icariin at LOQ (d-1), rat plasma sample obtained at 0.5 h after administration (c-2), and rat liver sample obtained at 0.5 h after administration (d-2).

In order to improve the peak shape and signal response of analytes and to reduce run time, different analytical columns and mobile phase compositions were tried to achieve good resolution and symmetric peak shape for icariin and IS. By comparison with Shiseido capcell pak C_18_ (150 × 2.0 mm i.d. 5 *μ*m) column, Agilent XDB C_18_ (150 × 2.1 mm i.d. 5 *μ*m) column could obtain better chromatographic behavior and higher signal response for the two analytes. Thus, this column was finally selected for chromatographic analysis. The modifier of the mobile phase composed of acetonitrile-water binary solvent system was screened among ammonium acetate, acetic acid, and formic acid. As a result, the mobile phase consisting of acetonitrile-water containing 0.1% formic acid could improve the symmetry of peak shape and enhance the signal response. The flow rate of mobile phase was also optimized and finally set at 0.3 mL/min. Under the above-mentioned HPLC conditions, the retention time of icariin and IS was within 6 min, and no interfering substance was detected at the retention time of analytes in blank rat plasma and tissue samples (Figure 3).

### 3.3. Method Validation

#### 3.3.1. Specificity

The selectivity of the method was examined by comparing the chromatograms of blank and corresponding spiked biological matrices. The retention time of icariin and IS was about 2.6 and 5.0 min, respectively. No interfering peak of endogenous substances was observed at the measured mass transitions and retention time of icariin and IS in blank biological matrix samples.

#### 3.3.2. Linearity and Sensitivity

The assay was found to be linear over the concentration range of 1–500 ng/mL for plasma and tissue homogenate. The mean linear regression equations were listed in Table 1, with correlations coefficient over 0.99, in which *X* corresponds to nominal concentrations and *Y* corresponds to peak area ratios. LLOQs with an *S*/*N* ratio of >20 was 1 ng/mL, which was sensitive enough for the pharmacokinetic and tissue distribution studies of icariin.

#### 3.3.3. Precision and Accuracy

Inter- and intra-assay variability at three different concentrations, HQC (400 ng/mL), MQC (20 ng/mL), LQC (2 ng/mL) was determined in plasma and tissue homogenate with five replicates on each day for three separate days. The detailed results of the inter- and intraday precision and accuracy of icariin in biological matrix were summarized in Table 2.

#### 3.3.4. Recovery and Matrix Effects

As shown in Table 3, the extraction recovery of icariin was in the range of 82.5–100.6% which suggested that the recovery of this method was consistent and reproducible. And the suppression of ionization in plasma and tissue homogenate was different. The interference of ionization caused by kidney homogenate was significant while that of brain homogenate was much lower.

#### 3.3.5. Stability

Five replicates of QC at three different concentration levels were used to assess the stability of icariin under various conditions. The results were summarized in Table 4, which indicated that icariin was stable in autosampler (24 h) at 4°C, bench-top (6 h) at room temperature, three freeze-thaw cycles, and frozen condition at −80°C for 30 days, as the RE values were within ±15% for the low, medium, and high concentrations.

#### 3.3.6. Plasma Pharmacokinetics

**Figure 4:**
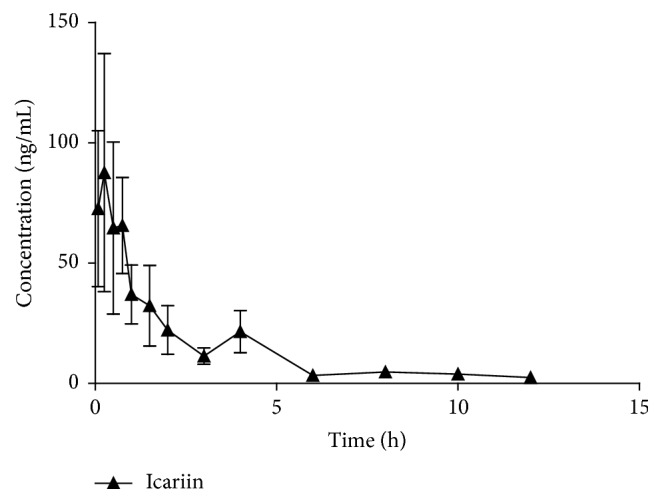
Plasma concentration versus time profile of icariin in rats (*n* = 6) following a single oral dose of Herba Epimedii extract (corresponding 42 mg/g icariin).

After a single oral administration of the total flavonoid extract of Herba Epimedii, the concentration of icariin in plasma was successfully determined by LC-MS/MS method described above. The mean plasma concentration-time profile of icariin was shown in Figure 4. Pharmacokinetic parameters were estimated using the DAS software and performed by noncompartmental analysis. Peak plasma concentration (*C*_max_) and the time at which it occurred (*T*_max_) were obtained from the visual inspection of the plasma concentration-time curve. Elimination rate constant (*K*_*e*_) was determined from the least-square fitted terminal log-linear portion of the plasma concentration-time profile. Elimination half-life (*t*_1/2_) was calculated by 0.693/*K*_*e*_. Total area under the plasma concentration-time curve of icariin, from time zero to infinity (AUC_0–*∞*_), was calculated as the sum of AUC_0–12_, the area up to the last quantifiable time point (12 h), and the area from the 12 h time point to infinity. AUC_0–12_ was calculated using the trapezoidal rule, and the extrapolated area was calculated from the last measurable concentration *C*_*t*_ divided by *K*_*e*_. The major pharmacokinetic parameters of icariin were summarized in Table 5.

### 3.4. Tissue Distribution

**Figure 5:**
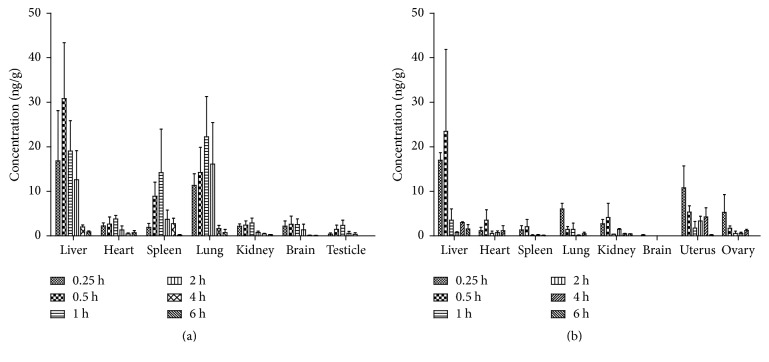
Tissue distribution of icariin in rats after a single oral dose of Herba Epimedii extract (corresponding 42 mg/g icariin, (a) male rat, (b) female rat).

The tissue distribution of icariin in female and male rats was investigated following a single oral dose of total flavonoid extract from Herba Epimedii (corresponding 42 mg/g icariin). The mean concentration-time profile of each tissue was presented in Figure 5, indicating that icariin was poorly absorbed after oral administration because all the tissue concentrations were kept at a very low level. The peak concentration of icariin in most of collected tissues appeared at 1 h after administration, except for liver and female reproductive organs, in which the concentration reached the peak at 0.5 h, demonstrating that icariin underwent a rapid and wide distribution. Moreover, the tissue concentrations in male rat were generally much higher than those in female rat. The tissue area under the curve from time zero to infinity was in the following order: liver > lung > spleen > heart > kidney > brain > testicle for mail rat, and liver > uterus > heart > ovary > lung > kidney > spleen > brain for female rats. As shown in Figure 5, the concentration levels in liver and lung were much higher than those in other tissues, illustrating that liver and lung are the main target organs of icariin in male rat after oral administration of the total flavonoid extract. Furthermore, the concentration level in uterus was much higher than that in any other tissues of female rat, demonstrating that the gender-related difference of icariin in tissue distribution do exist and the reproductive organs are the main target organs of icariin for female rat. Meanwhile, icariin was found with very low AUC_0–*∞*_ in brain, which indicated that icariin could not efficiently cross the blood-brain barrier. Over time, the main trend of the mean concentration of icariin in all tissues was reduced, and the concentration dropped very quickly at 4 h after administration, suggesting no accumulation in tissues and a rapid elimination of this compound.

### 3.5. Metabolism Study of Icariin in Rat Urine and Feces

#### 3.5.1. Metabolite Profile of Rat Urine and Feces

**Figure 6:**
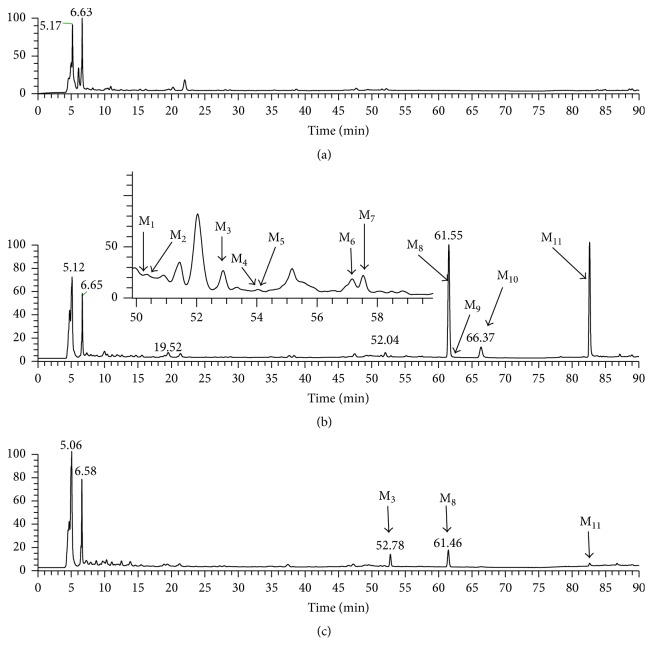
Representative chromatograms of icariin metabolites in rat feces of 0–24 h: (a) blank feces sample; (b) feces sample after oral administration; and (c) feces sample after intramuscular administration.

**Figure 7:**
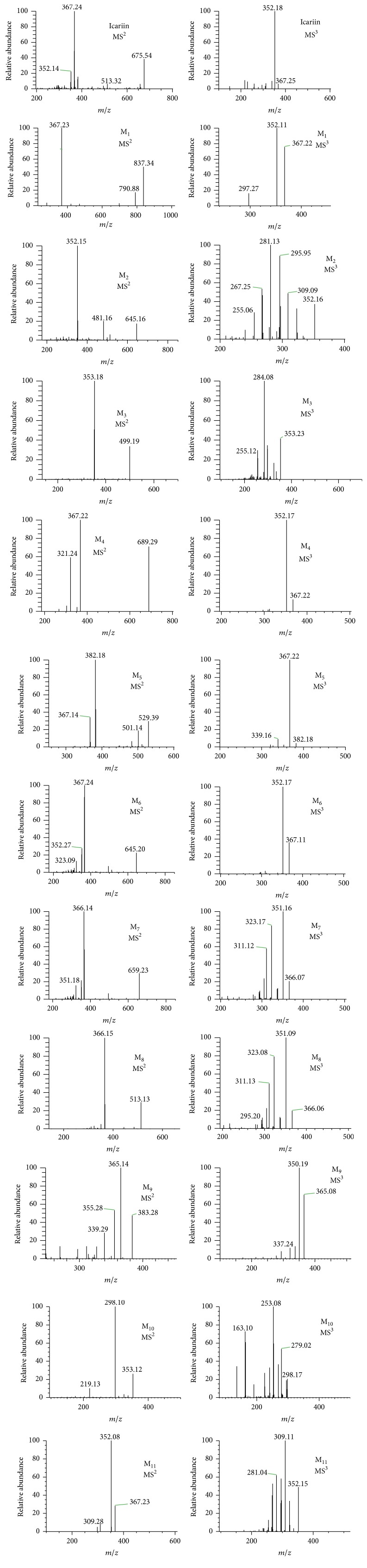
Representative LC-MS^n^ spectra of icariin and its metabolites M_1_–M_11_ in rat feces.

**Figure 8:**
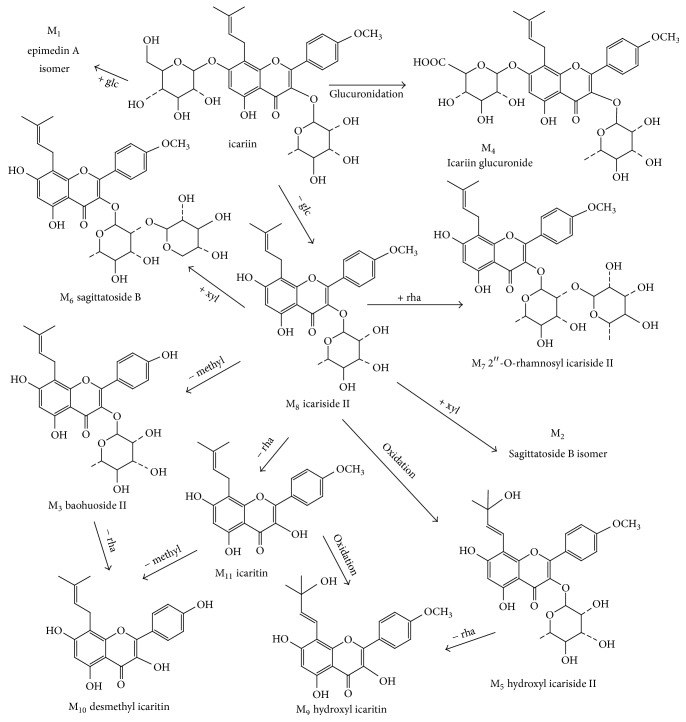
Proposed metabolic pathways for icariin in rat feces.

In this study, urine and feces samples were both analyzed by LC-MS/MS. By comparing the total ion chromatograms of drug-containing urine sample and predose control urine sample, neither parent drug nor metabolites were observed in rat urine following two routes of administration of icariin. However, a total of 11 icariin metabolites were detected in rat feces, including 10 metabolites after oral administration and 9 following intramuscular introduction. The representative chromatograms of the metabolites of 0–24 h rat feces were shown in Figure 6. No additional metabolites were observed in the feces of 24–48 h collection period. Results showed that the major metabolites of icariin were M_8_ and M_11_, whose total amount accounted for over 90% of total peak area in the LC-UV chromatogram of 0–24 h feces. And the concentration of metabolites in feces rapidly declined over 24–48 h collection period. The MS^2^ and MS^3^ spectra of icariin and its metabolites were exhibited in Figure 7, and the observed and calculated masses, formula, retention times, mass errors, and MS data of these analytes were presented in Table 6. Proposed metabolic pathways for icariin in rat feces were shown in Figure 8.

#### 3.5.2. Identification of Metabolites

Known compounds were identified by comparison with the chromatographic behavior and MS fragmentation pattern of reference standards. For unknown constituents, their structures were characterized based on their retention behavior and their MS^n^ spectra obtained on-line. Moreover, under the optimized LC-MS conditions, all the in vivo metabolites of icariin could give the precursor ions of sufficient abundance in order to facilitate the analysis of multistage mass spectrometry. Because all metabolites originated from icariin and their MS fragmentation patterns were largely similar to that of icariin, it was necessary to characterize the MS^n^ behavior of icariin to identify and assign the metabolites of the constituent. The deprotonated molecule [M–H]^−^ of icariin appears at *m*/*z* 675, which readily eliminates its glucosyl and rhamnosyl groups to produce an [M–H–308]^−^ ion at *m*/*z* 367. The [M–H–308]^−^ ion yields a fragment at *m*/*z* 352 by losing a methyl radical. And of the MS^4^ spectrum of [M–H–308–15]^−^ ion, the fragment at *m*/*z* 323 can be reasonably attributed to the loss of 3-COH of flavone skeleton's C ring.

M_1_, as a minor metabolite of icariin, was only found in the feces of the oral group. Its quasimolecular ion occurs at *m*/*z* 837 (C_39_H_49_O_20_), whose molecular mass is 162 Da more than that of icariin. And its major MS^n^ fragment ions are the same as those of icariin. The most abundant MS^2^ and MS^3^ fragment ions also appear at *m*/*z* 367 and 352, indicating the loss of one rhamnosyl and two glucosyl moieties and the sequential loss of methyl group, respectively. Although the molecular weight of M_1_ is equal to that of epimedin A, there exists a significant difference on their chromatographic retention behavior. Therefore, M_1_ is tentatively identified as an isomer of epimedin A.

Both M_2_ and M_6_ show the same quasimolecular ion [M − H]^−^ at *m*/*z* 645 (C_32_H_37_O_14_) with the elution time at 50.44 and 57.43 min, respectively. M_2_ was only observed in feces after intramuscular introduction of icariin, while M_6_ was found in feces following both routes of administration. M_6_ is unambiguously identified as sagittatoside B in comparison with the reference standard. Analogous to the MS^n^ fragments of icariin (see Figure 7 and Table 6), M_2_ also yielded the major fragment ions at *m*/*z* 352, 323, 309, 296, 281, and 268, strongly suggesting that M_2_ should be a conjunction metabolite of icariin. Thus, M_2_ is tentatively assigned as sagittatoside B isomer.

M_3_ was found in rat feces after both intramuscular and oral administration of icariin. Its MS spectrum exhibits a deprotonated molecule [M–H]^−^ at *m*/*z* 499 (C_26_H_27_O_10_), 176 Da less than that of icariin. By comparison with the reference standard, it is identified as baohuoside II. The MS/MS spectrum shows the most abundant fragment ion at *m*/*z* 353, indicating the loss of rhamnose moiety. The MS^3^ spectrum displays the major fragment ions at *m*/*z* 325, 298, and 284, separately corresponding to the loss of CO, isobutenyl, and isopentenyl radicals.

M_4_ is a minor metabolite of icariin detected in the feces of rats in the oral administration group. Its deprotonated molecule (C_33_H_37_O_16_) is at *m*/*z* 689. And the major MS/MS fragment at *m*/*z* 367 results from the loss of glucuronic acid and rhamnose moieties. Its MS^3^ spectrum produces a fragment at *m*/*z* 352 (the loss of a methyl group). Thus M_4_ is tentatively characterized as icariin glucuronide.

M_5_–M_11_ of icariin metabolites were observed in rat feces after both intramuscular and oral administration of icariin.

M_5_ has a deprotonated molecular ion at *m*/*z* 529 (C_27_H_29_O_11_) in its MS spectrum. The MS^n^ spectra show the major fragment ions at *m*/*z* 501 (the loss of CO), 382 (the loss of rhamnosyl group), 367 (the loss of methyl radical from the ion at *m*/*z* 382), and 339 (the successive loss of methyl radical and CO). Therefore, M_5_ is tentatively identified as hydroxyl icariside II.

M_7_ is identified as 2′′-O-rhamnosyl icariside II based on the reference standard and related mass spectral pattern. The MS spectrum of M_7_ shows a quasimolecular ion [M–H]^−^ at *m*/*z* 659 (C_33_H_39_O_14_). The major fragment ions at *m*/*z* 366, 351, and 323 result from the concurrent loss of the rhamnose chain, the successive loss of a methyl group, and CO, respectively.

As shown Figure 7, M_8_ is the most abundant metabolite of icariin. By comparison with the reference standard, it is identified as icariside II. Its MS spectrum shows a deprotonated molecular ion at *m*/*z* 513 (C_27_H_29_O_10_). And similar to the MS^n^ pattern of icariin, the major fragment ions at *m*/*z* 366, 351, and 323 separately match the loss of rhamnose moiety, the sequential loss of rhamnose and methyl groups, and the loss of CO.

M_9_ has a quasimolecular ion [M–H]^−^ at *m*/*z* 383 (C_21_H_19_O_7_), 146 Da less than that of M_5_ (hydroxyl icariside II). It is tentatively identified as hydroxyl icaritin. Its MS/MS spectrum shows the main fragments of *m*/*z* 365, 355, and 339, respectively, corresponding to the neutral loss of H_2_O, CO, and CO_2_. MS^3^ spectrum, the fragment ions at *m*/*z* 350 and 321 individually result from the neutral loss of methyl radical and CO_2_.

The quasimolecular ion [M–H]^−^ of M_10_ is at *m*/*z* 353, 146 Da less than that of M_3_ (baohuoside II). The major fragment ion in its MS/MS spectrum is at *m*/*z* 298, matching the loss of isobutenyl radical. The fragment at *m*/*z* 219 is possibly produced by the neutral loss of C_8_H_6_O_2_ (134 Da) due to the retro-Diels-Alder (RDA) fragmentation of the flavone's C ring. Moreover, the MS^3^ spectrum affords fragment ions at *m*/*z* 279, 269, 253, and 164, resulting from the loss of H_2_O, CO, CO_2_, and C_8_H_6_O_2_ (134 Da), respectively. Thus this metabolite is reasonably identified as desmethyl icaritin.

M_11_ was observed as another major metabolite in rat feces of both two administration groups (Figure 7). By comparison with the reference standard, it is identified as icaritin. Its MS spectrum yields a quasimolecular ion at *m*/*z* 367 (C_21_H_19_O_6_). And the major fragment ion at *m*/*z* 352 is characterized as the loss of methyl group. The MS^3^ fragment at *m*/*z* 309 results from the loss of methyl group and CO, and ion at *m*/*z* 297 is the outcome of the neutral loss of isopentene (70 Da) from the deprotonated molecule.

## 4. Conclusion

The menopausal syndrome improvement and possible cancer preventive activity of Herba Epimedii is receiving more and more attention. Information on the in vivo disposition of Herba Epimedii components is important for better understanding the biological effects of this herbal drug. For this purpose, our investigation was designed to obtain information on the plasma pharmacokinetics and tissue distribution of icariin in rat and the excretion of icariin and its metabolites. In this study, we firstly established and validated a rapid, sensitive, and reliable LC-MS method for the determination of icariin in various biological samples collected at different time points, and then the method was applied to the plasma pharmacokinetics and tissue distribution study of icariin in rats after oral administration of the total flavonoid extract of Herba Epimedii. In addition, the differences of the tissue distribution of icariin in between male and female rats were studied after oral and intramuscular administration of icariin. And the metabolic pattern of icariin was also investigated. The data showed the plasma and tissue concentration of icariin were kept at a very low level at all collection points, which suggests icariin is poorly absorbed after oral administration. The peak concentration in plasma and collected tissue samples appeared at 0.5–1 h and then quickly dropped at 4 h after administration, demonstrating that a small amount of icariin can be rapidly absorbed and eliminated, and there is no accumulation in rat tissues. Moreover, the concentration in liver, lung, and reproductive organs was much higher than that in other tissues, indicating that liver, lung, and reproductive organs are the main target sites of icariin in rats.

The gender-related difference of icariin in tissue distribution has not been reported in the past published literature. Our study demonstrated that the distribution features of icariin are similar in most of tissues between male and female rats. However, there exist a few remarkable gender differences, such that the whole tissue concentration of icariin in male rats was much higher than female rats, except in genital organs (high in female and low in male). In China, Herba Epimedii is a famous tonic and aphrodisiac to invigorate kidney and strengthen Yang; therefore, it is commonly used to treat male sexual functional decline and female menopausal syndrome. As one of the predominant constitutes in Herba Epimedii, icariin plays a very crucial role in exerting effects, so the relatively high icariin concentration in female genital organs actually supports the above-mentioned traditional Chinese medicine theory and clinical practice and makes this compound a good future prospect for treating women's menopausal related diseases.

In the present study, the complete metabolic profile of icariin has also been investigated. The results illustrate that feces should be the major excretion way for both oral and intramuscular administration of icariin. Based on LC-UV and LC-MS^n^ analyses, eleven metabolites were detected in fecal samples and were further identified. Among them, 10 metabolites appeared in rat feces of oral administration group and 9 in the feces of intramuscular introduction group. The major metabolic routes of clearance are proposed to be hydrolysis, demethylation, oxidation, and conjugation.

## Figures and Tables

**Table 1:** Regression equations of icariin in rat plasma and tissue samples (*n* = 3).

Sample	Equation	Correlation coefficient	Linear range
			ng/mL
Plasma	*Y* = (0.0060 ± 0.0002)*X* + (0.0011 ± 0.0006)	0.9973 ± 0.0013	1.00–500
Liver	*Y* = (0.0053 ± 0.0001)*X* + (0.0014 ± 0.0008)	0.9986 ± 0.0008	1.00–500
Heart	*Y* = (0.0062 ± 0.0003)*X* + (0.0006 ± 0.0002)	0.9979 ± 0.0013	1.00–500
Spleen	*Y* = (0.0053 ± 0.0006)*X* + (0.0010 ± 0.0008)	0.9979 ± 0.0009	1.00–500
Lung	*Y* = (0.0060 ± 0.0006)*X* + (0.0015 ± 0.0008)	0.9974 ± 0.0015	1.00–500
Kidney	*Y* = (0.0045 ± 0.0003)*X* + (0.0006 ± 0.0002)	0.9986 ± 0.0004	1.00–500
Brain	*Y* = (0.0033 ± 0.0002)*X* + (0.0011 ± 0.0003)	0.9985 ± 0.0005	1.00–500
Testicle	*Y* = (0.0066 ± 0.0001)*X* + (0.0015 ± 0.0001)	0.9977 ± 0.0001	1.00–500

**Table 2:** Intra- and interday accuracy and precision for the determination of icariin in plasma and tissue samples (*n* = 18, 6 replicates per day for 3 days).

c	Conc.	Interday		Intraday	
	ng/mL	RE (%)	CV (%)	RE (%)	CV (%)
Plasma	2	−0.394	13.4	−0.887	3.50
	20	−5.18	1.56	−4.85	2.25
	400	−3.28	1.50	−2.76	2.47

Liver	2	0.690	1.55	−0.197	4.64
	20	−0.528	9.59	−0.198	1.85
	400	−1.03	10.4	−1.17	1.62

Heart	2	−0.309	4.25	−4.83	3.22
	20	4.09	1.61	4.95	2.46
	400	−3.65	2.19	−2.57	1.59

Spleen	2	−2.14	9.00	−3.35	7.63
	20	3.96	5.09	3.46	2.25
	400	−0.313	4.75	0	1.98

Lung	2	−6.80	13.7	−13.3	4.72
	20	−2.05	14.1	−8.52	2.67
	400	−9.30	10.2	−1.26	1.11

Kidney	2	4.73	5.36	3.94	5.36
	20	3.99	13.3	7.43	2.77
	400	−6.49	13.6	−5.73	1.49

Brain	2	−13.1	9.48	−12.3	4.74
	20	3.27	5.59	0.792	1.40
	400	−3.28	3.56	−3.36	2.12

Testicle	2	−4.20	3.25	−2.36	4.60
	20	0.538	4.93	3.07	2.69
	400	−0.379	5.79	2.57	4.02

**Table 3:** Matrix effect and extraction recovery for the assay of icariin in plasma and tissue samples (*n* = 5).

Matrix	Conc. ng/mL	Matrix effect Mean ± SD (%)	Extraction efficiency Mean ± SD (%)
Plasma	2	71.2 ± 2.9	86.9 ± 6.0
	20	69.0 ± 2.0	84.1 ± 4.7
	400	66.7 ± 1.0	84.9 ± 5.8

Liver	2	71.5 ± 3.3	93.9 ± 7.5
	20	72.2 ± 4.1	90.3 ± 1.9
	400	70.3 ± 3.1	90.5 ± 2.2

Heart	2	67.9 ± 3.8	88.9 ± 3.9
	20	68.8 ± 2.6	92.2 ± 2.3
	400	69.9 ± 1.8	93.1 ± 1.7

Spleen	2	69.2 ± 5.3	88.7 ± 8.4
	20	70.1 ± 3.7	83.5 ± 3.9
	400	71.4 ± 3.8	82.5 ± 1.4

Lung	2	71.0 ± 2.2	88.0 ± 5.9
	20	71.2 ± 2.4	85.4 ± 4.5
	400	73.8 ± 1.6	83.8 ± 1.9

Kidney	2	57.7 ± 6.2	86.5 ± 6.7
	20	55.5 ± 5.3	90.0 ± 3.7
	400	66.6 ± 15.2	85.3 ± 6.1

Brain	2	90.6 ± 5.8	89.7 ± 5.7
	20	89.3 ± 1.9	94.9 ± 3.9
	400	87.0 ± 2.3	99.0 ± 2.3

Testicle	2	80.2 ± 3.5	91.5 ± 6.7
	20	77.3 ± 2.1	96.4 ± 2.2
	400	77.7 ± 2.2	100.6 ± 1.9

**Table 4:** Stability of icariin in plasma and tissue samples (*n* = 6).

Matrix	Conc.	Mean ± SD			
	ng/mL	Bench-top^a^	Freeze-thaw^b^	Autosampler^c^	Long-term^d^
Plasma	2	97.5 ± 3.7	96.8 ± 5.7	104.7 ± 8.1	99.3 ± 5.9
	20	94.5 ± 5.9	101.3 ± 3.1	95.5 ± 3.7	94.3 ± 1.6
	400	97.7 ± 3.9	103.0 ± 1.9	94.5 ± 2.6	95.5 ± 3.7

Liver	2	98.7 ± 6.3	101.5 ± 2.1	111.4 ± 4.4	98.6 ± 5.4
	20	94.9 ± 1.4	90.0 ± 1.3	106.0 ± 1.6	95.7 ± 2.1
	400	87.5 ± 1.3	89.7 ± 1.3	89.4 ± 1.4	112.5 ± 2.2

Heart	2	108.6 ± 3.3	100.2 ± 4.3	95.5 ± 2.2	115.0 ± 3.0
	20	105.6 ± 1.7	99.6 ± 1.0	97.3 ± 1.3	100.2 ± 4.2
	400	102.6 ± 1.5	94.2 ± 2.9	89.2 ± 0.9	90.7 ± 5.0

Spleen	2	95.4 ± 9.2	101.8 ± 4.4	95.5 ± 7.6	101.3 ± 5.9
	20	98.9 ± 1.4	109.4 ± 1.1	92.8 ± 0.9	112.3 ± 3.4
	400	96.7 ± 2.7	103.2 ± 0.4	87.1 ± 1.2	97.4 ± 2.6

Lung	2	95.9 ± 3.5	85.6 ± 3.0	103.2 ± 4.8	98.3 ± 4.3
	20	101.7 ± 8.0	92.3 ± 4.0	109.4 ± 4.6	110.2 ± 1.8
	400	92.5 ± 1.8	93.2 ± 1.5	99.7 ± 0.4	109.8 ± 1.7

Kidney	2	101.2 ± 3.4	91.6 ± 4.0	95.1 ± 2.2	98.4 ± 4.8
	20	102.5 ± 2.8	97.4 ± 1.8	95.5 ± 1.6	93.4 ± 2.2
	400	92.0 ± 1.4	91.1 ± 0.8	89.9 ± 1.0	109.5 ± 2.2

Brain	2	85.8 ± 6.0	97.4 ± 7.3	96.6 ± 3.2	109.4 ± 3.2
	20	103.3 ± 4.1	103.5 ± 3.1	103.0 ± 3.5	97.4 ± 1.2
	400	100.2 ± 2.9	99.0 ± 3.1	97.8 ± 2.1	110.2 ± 3.0

Testicle	2	105.8 ± 3.9	85.3 ± 2.4	99.7 ± 5.5	93.3 ± 4.1
	20	104.0 ± 1.4	87.0 ± 1.3	101.6 ± 1.9	106.5 ± 1.5
	400	103.4 ± 3.9	98.4 ± 1.6	99.5 ± 3.3	106.8 ± 3.8

^a^At least 1 h at room temperature. ^b^At least 3 freeze-thaw cycles. ^c^At least 24 h at 4°C. ^d^At least 4 weeks at −80 ± 5°C.

**Table 5:** Pharmacokinetic parameters of icariin in rat after a single oral dose of Herba Epimedii extract (corresponding 42 mg/g icariin).

Parameter	Icariin
AUC_0–*∞*_ (ug/L·h)	164.155 ± 144.129
AUMC_0–*∞*_ (ug/L·h)	538.424 ± 262.081
MRT_0–*∞*_ (h)	3.990 ± 1.987
*t*_1/2_ (h)	3.149 ± 2.364
*T*_max_ (h)	0.458 ± 0.246
CL_*z*_/*F* (L/h/kg)	389.043 ± 204.472

**Table 6:** Characterization of metabolites of icariin in rat feces.

Number	Compound	RT	Formula	Observed mass	RDB	Error	LC/MS^n^ data
				[M–H]^−^		(ppm)	
P^*∗*^	Icariin	55.19	C_33_H_39_O_15_	675.22888	14.5	0.790	MS^2^ [675] 367 (100), 352 (25) MS^3^ [675-367] 352 (100) MS^4^ [675-367-352] 323 (30), 309 (100), 296 (45), 281 (65), 268 (50), 255 (20)

M_1_	Epimedin A isomer^a^	50.38	C_39_H_49_O_20_	837.2819	15.5	0.824	MS^2^ [837] 790 (50), 367 (100) MS^3^ [837-367] 352 (100), 297 (20)

M_2_	Sagittatoside B isomer^b^	50.44	C_32_H_37_O_14_	645.2186	14.5	1.314	MS^2^ [645] 481 (20), 352 (100) MS^3^ [645-352] 323 (40), 309 (60), 296 (90), 281 (100), 268 (60)

M_3_^ ^^*∗*^	Baohuoside II^a,b^	52.78	C_26_H_27_O_10_	499.1605	13.5	1.295	MS^2^ [499] 353 (100) MS^3^ [499-353] 325 (20), 298 (40), 284 (100), 255 (30)

M_4_	Icariin glucuronide^a^	54.04	C_33_H_37_O_16_	689.2084	15.5	1.202	MS^2^ [689] 367 (100), 321 (60) MS^3^ [689-367] 352 (100)

M_5_	Hydroxyl icariside II^a,b^	54.12	C_27_H_29_O_11_	529.1716	13.5	1.132	MS^2^ [529] 501 (20), 382 (100), 367 (40) MS^3^ [529-382] 367 (100), 339 (10)

M_6_^ ^^*∗*^	Sagittatoside B^a,b^	57.43	C_32_H_37_O_14_	645.2184	14.5	1.020	MS^2^ [645] 367 (100), 352 (30) MS^3^ [645-367] 352 (100)

M_7_^ ^^*∗*^	2′′-O-Rhamnosyl-icariside II^a,b^	57.56	C_33_H_39_O_14_	659.2344	14.5	1.529	MS^2^ [659] 366 (100), 351 (20), 323 (15) MS^3^ [659-366] 351 (100), 323 (80), 311 (60), 305 (20)

M_8_^ ^^*∗*^	Icariside II^a,b^	61.46	C_27_H_29_O_10_	513.1760	13.5	0.851	MS^2^ [513] 366 (100) MS^3^ [513-366] 351 (100), 323 (80), 311 (50), 305 (20)

M_9_	Hydroxyl icaritin^a,b^	62.33	C_21_H_19_O_7_	383.1137	12.5	3.160	MS^2^ [383] 365 (100), 355 (60), 339 (40) MS^3^ [383-365] 350 (100), 321 (20), 230 (40)

M_10_	Desmethyl icaritin^a,b^	66.36	C_20_H_17_O_6_	353.1024	12.5	1.119	MS^2^ [353] 298 (100), 219 (10) MS^3^ [353-298] 279 (60), 269 (40), 253 (100), 241 (30), 164 (70)

M_11_^ ^^*∗*^	Icaritin^a,b^	82.65	C_21_H_19_O_6_	367.1183	12.5	1.921	MS^2^ [367] 352 (100), 309 (20), 297 (20) MS^3^ [367-352] 309 (100), 297 (60)

^*∗*^Compared with the reference standard. ^a^Observed in rat feces after oral administration. ^b^Observed in rat feces after intramuscular administration.
